# Systematic Review and Critical Analysis of Cost Studies Associated with Parkinson's Disease

**DOI:** 10.1155/2017/3410946

**Published:** 2017-03-05

**Authors:** Tânia M. Bovolenta, Sônia Maria Cesar de Azevedo Silva, Roberta Arb Saba, Vanderci Borges, Henrique Ballalai Ferraz, Andre C. Felicio

**Affiliations:** ^1^Programa de Pós-Graduação, Hospital Israelita Albert Einstein, São Paulo, SP, Brazil; ^2^Movement Disorders Department in Neurology, Universidade Federal de São Paulo (UNIFESP), São Paulo, SP, Brazil; ^3^Universidade Federal de São Paulo (UNIFESP), São Paulo, SP, Brazil; ^4^Neurology Program, Hospital Israelita Albert Einstein, São Paulo, SP, Brazil

## Abstract

Parkinson's disease (PD) is the second most prevalent neurodegenerative disease worldwide, affecting more than four million people. Typically, it affects individuals above 45, when they are still productive, compromising both aging and quality of life. Therefore, the cost of the disease must be identified, so that the use of resources can be rational and efficient. Additionally, in Brazil, there is a lack of research on the costs of neurodegenerative diseases, such as PD, a gap addressed in this study. This systematic review critically addresses the various methodologies used in original research around the world in the last decade on the subject, showing that costs are hardly comparable. Nonetheless, the economic and social impacts are implicit, and important information for public health agents is provided.

## 1. Introduction

Health and the economy are related intrinsically. The purpose of the studies on the costs of diseases is describing them, estimating costs, comparing established programs, and projecting these costs based on clinical, demographic, epidemiological, and technological factors. In fact, over the past decade, there has been a growing number of studies, which are presumed to be valuable decision tools, because the limited amount of resources must be used rationally and efficiently as not to miss opportunities to improve overall population health [1].

In neurodegenerative diseases, such as Parkinson's disease (PD), whose prevention is still impossible, the burden borne by society, whether it is financial, social, or even psychological, is often heavy. Being the second most prevalent neurodegenerative disease worldwide, it generally affects individuals between 40 and 50 (late-onset PD) [2, 3], compromising their productive life and aging. As such, research needs to be directed to reducing their costs.

Studies on disease costs may have several approaches, such as economic assessment, epidemiological design, or even type of cost involved, as well as the viewpoint of defining resource use strategies. The diversity in methods is a significant factor why cost estimates differ between studies, opening the discussion about which public policies are most appropriate for PD.

This systematic review provides introductory concepts on the types of studies on costs and analyzes results in selected articles critically, highlighting the benefits and limitations of their methods. Moreover, this study identifies the most common studies regarding DP costs worldwide over the past 10 years, showing possibilities for studies being carried out in Brazil, where there is a lack of this type of analysis because most studies only involve the clinical aspects of the disease.

## 2. Methodology

In March 2016, two* online* bibliographic information services were accessed—SCOPUS and PubMed—with the aim of selecting original articles about the cost of PD over the past decade.

The following terms were used for access:* (parkinson disease) AND TITLE-ABS-KEY (economics) OR TITLE-ABS-KEY (costs of illness) OR TITLE-ABS-KEY (health expenditures) OR TITLE-ABS-KEY (cost effectiveness analysis) OR TITLE-ABS-KEY (cost benefit analysis) OR TITLE-ABS* - CUT (cost utility analysis) OR TITLE-ABS-KEY (cost minimization analysis) OR TITLE-ABS-KEY (direct costs) OR TITLE-ABS-KEY (CB costs) OR TITLE-ABS-KEY (out of pockets) AND DOCTYPE (air OR re) AND PUBYEAR > 2004 AND (LIMIT-TO (LANGUAGE, “English”)) AND (LIMIT-TO (DOCTYPE, “air”)).

**Figure 1:**
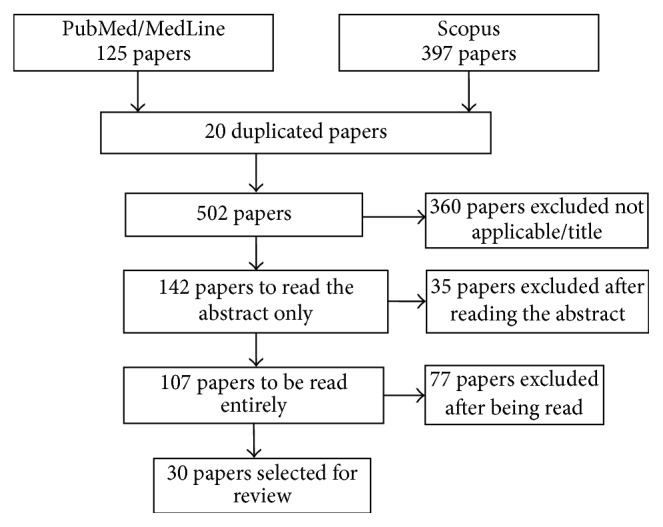
Search, selection, and inclusion of papers for critical analysis of studies on economic PD evaluation in* online* platforms.

This method identified 522 papers. The inclusion criterion was that articles refer to costs related to this disease in general and/or regarding the use of medication. Papers that compared procedures and/or medicines, dealt with specific therapies, as well as PD surgeries, or were related to patient caregivers or already selected papers but were neither applicable to the research nor available to access were excluded. Revisions were also not selected (380 papers were excluded because of the title, for not being compatible, having been duplicated, and/or being reviews). Once the first levels of inclusion were satisfied, 142 papers were selected for reading the* abstract*. Although 35 of these had a suggestive title, that is, they did not tackle general costs of disease exclusively, 107 were separated for complete reading, and 30 met the criteria for research (Figure 1).

### 2.1. Basic Concepts of Health Studies

The determination of the costs of a disease facilitates learning what its burden to society is, assessing its degree of efficiency, and understanding how the market tends to organize itself regarding certain values [4].

#### 2.1.1. Economic Assessment

The basic function of any economic assessment is to identify, measure, value, and compare the costs and consequences of alternative proposals [4–8].

In this case, four techniques are possible (Table 1):

*(1) Cost-Minimization.* Cost-minimization is the least used technique, because it only compares costs of interventions that produce the same outcomes with different costs. For chronic diseases, such as PD, there are no studies using this type of analysis.

*(2) Cost-Effectiveness.* Cost-effectiveness is the technique most used in literature, which assesses the impact of different alternatives that bring better results with lower costs; these are always comparative and explicit and designed to select the best option to achieve what is perceived in clinical practice.

*(3) Cost-Benefit.* This analysis determines whether a new health technology or intervention generates net benefits to society. However, due to its difficulty, complexity, and controversies in valuing human life and certain health conditions in monetary terms, this analysis is rarely found in the literature.

*(4) Cost-Utility.* This analysis assesses the impacts on survival and quality of life, which are determining criteria to judge the effects of strategies in health care, that is, the level of well-being and preferences of the individual.

#### 2.1.2. Study Designs

The epidemiological study designs define how the research will be performed in relation to adopted method [9–13]. The most discussed study designs in PD research are as follows (Table 2).

*(1) Prevalence and Incidence.* The prevalence estimates the number of deaths, hospitalizations, prevention, and research attributable to a disease in a given period (usually a year), to subsequently estimate the costs incurred by these consequences. Incidence refers to the number of new cases in a predefined period, and it foresees the associated costs from the onset of the disease until its disappearance (usually cure or death), through a rough projection of the flow of these values.

Studies on prevalence display greater results than incidence ones, for diseases are usually long-term sequelae, as is the case of PD, and they are of great importance in planning specific policies on certain diseases when their economic burden was underestimated. Therefore, they can identify the main components of current expenses and uncharged resources.

*(2) Top-Down and Bottom-Up.  Top-down* approaches are normally used in prevalence studies, when the expenses of a disease are widely known from national or regional statistics. In* bottom-up* studies, cost estimates are more detailed. The data depend on the scope of the study, and they are intrinsically related to the unit costs of inputs used, through interviews, questionnaires or chart review, and assessment of individual cost. The average cost per person is then obtained by means of the number of times the service was used and the number of people with the disease. Although* bottom-up* studies are more complete when it comes to resources and more precise regarding patient selection, they run a high risk of double counting costs (e.g., if a patient has more than one disease and costs of comorbidities are confused and/or grouped). The majority of studies with PD adopt this approach.

*(3) Prospective and Retrospective Approaches.* There is a temporal relationship, where in prospective studies the relevant events have not happened yet, that is, studying the patient over time, formalizing a system of data collection focused on the purpose of the research, such as questionnaires designed specifically for patients and/or their caregivers, where everything is recorded in “real time.” In retrospective studies, all events had already occurred when the study was initiated. They are usually employed in long-term chronic diseases, as is the case of PD. In this case, research efficiency can only be possible with enough observational datasets. It would be best if the data were stored electronically to minimize memory bias due to omission of facts or values.

*(4) Econometric Approaches.* Econometric approaches estimate differences between groups. One of the groups has the disease and the other does not; however, both have the same characteristics, which are assessed by several regression analyses involving demographic factors such as sex, age, marital status, ethnicity, relationship between patient and caregiver, housing, and duration of the disease.

*(5) Markov Models.* Markov models are used in several studies of chronic diseases, when patients are studied over time, and they are stratified according to disease scale. In the case of PD, the scale of Hoehn and Yahr (it assesses the degree of disability due to the disease in scores) is key to building this model. These are typically prospective studies, proposing cost increases with disease severity.

Several approaches may be featured in the same study; that is, we may have a retrospective, prevalent, and* bottom-up* study, for instance, because its purpose, most of the time, is to maximize the content of information, contributing to enriching knowledge.

#### 2.1.3. Classification of Costs

The costs of a disease are typically stratified as follows [4, 7, 10, 14] (Table 3).

*(1) Direct Costs.* Direct costs are related to the disease and its equation; their charges may concern public administration, insurance companies, the patient, the patient's family, or even a combination of all or some of these determinants. The estimates of direct costs associated with chronic diseases are higher than those associated with acute and communicable diseases, on the condition that better treatments and methods of prevention are adopted. This group can be divided into direct medical costs and nonmedical ones, although not all studies adopt this division.

*(2) Indirect Costs.* Indirect costs refer to the loss of income and/or productivity; they are caused by disease. Additionally, they can incur costs to both the patient and the employer. Depending on the disease, this loss may be partial, temporary, or permanent, and it may be restricted to the patient and/or caregiver (as in the case of advanced stage PD), frequently leading to early retirement. If there is a possibility of returning to regular activities, this disease may not occur on the same productivity level as before, or lead to frequent absences (absenteeism), incurring additional costs, such as loss of promotions.

*(3) Intangible Costs.* Intangible costs are virtually impossible to measure, since they incur psychological and psychosocial costs imposed to the patients, their family, and acquaintances due to the disease, as well as pain, behavioral changes, and everyday activities. They depend on the perception that the patient's health problems lead to social consequences, such as isolation.

*(4) Personal Costs.* Personal costs are the costs borne by the patient and/or their family and friends due to consultations with health professionals, medication, laboratory tests, domestic adjustments, locomotion resources, and the need for home care. Depending on the country, these costs, also called copayments, are borne by the government, health insurance, or religious or private health institutions. Sometimes, these payments are also designated as direct costs because they are associated with the disease. They may be redeemable or not, implying an additional expense to the patient, who has to spend a certain amount of money in advance.

#### 2.1.4. Perspectives

Who bears the costs related to the program defines which costs are included for analysis [6, 7, 10] (Table 4).

*(1) Industry (Human Capital).* The industry bears the costs due to absenteeism or loss of productivity, and early retirement due to the disease.

*(2) Society.* A more comprehensive perspective considers all costs related to the program, regardless of who will pay the expenses (patient, government, or insurance companies). This approach is thought to be the most appropriate to support health-related decisions. Most research on PD addresses this perspective, although some studies include more than one viewpoint.

*(3) Patients and Their Family.* Costs are borne by the patient concerning appointments, transport used for his treatment, purchase of medication, expenses with caregivers, domestic changes, and so on.

*(4) Programs, Public Health, and/or Insurance Companies.* When there is a need to identify all the inputs related to the disease, for which monetary value explains the base period and the form of assessment used should be assigned, this perspective is highly likely to underestimate the cost of disease, especially when greater profit or lower production costs are targeted.

### 2.2. Studies on the Socioeconomic Impact of PD

Although PD affects more than four million people worldwide [15], little is known about their progression rates, the costs of medical care, and the management of resources specific to this disease [16]. In Brazil, although its notification is not compulsory, unofficial data estimate 220,000 PD sufferers. Considering local records of patients with PD, in a study conducted in the city of Bambuí, Minas Gerais, it was found that 3% of the population above 64 had the disease, a result similar to the prevalence rates found in elderly studies in European and American countries and slightly above the rates in Eastern countries [17].

PD was considered among the most prevalent and costly diseases of the brain, being the fourth most expensive second study in 28 European countries [18]. However, the level of socioeconomic development, budget availability of health systems, and culture of each country or region determine research methodologies, which are directed to a subset of expenses, using only a few components and all expenditure resulting from a disease, which would be practically impossible. Therefore, there is no one method more or less appropriate for this type of study, as the costs of PD in all countries involved cannot be compared and the information cannot be simply transferred from one country to another without having any evidence to support the use of the data.

**Figure 2:**
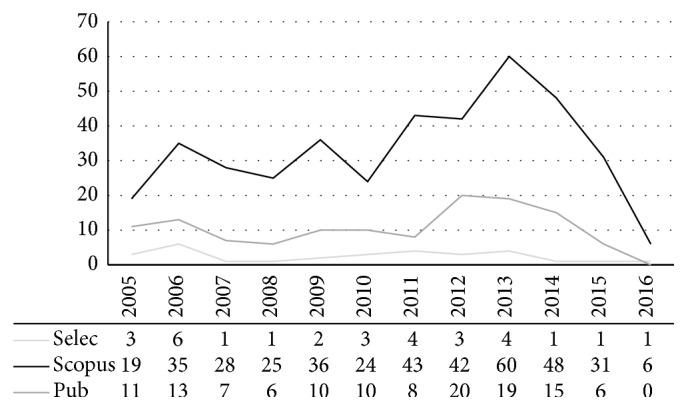
Number of publications on PD costs over the past 10 years. Scopus = 397 papers; Pubmed = 125 papers; Selected = 30.

Over the past decade, there has been a significant increase in the number of papers related to costs of diseases.  Figure 2 shows the evolution for PD, between 2005 and February 2016, with a higher concentration between 2011 and 2013, triggered by the need to investigate the values involved in the cost of this disease. The demographic transition is a reality, and health managers need data that can enable their strategies towards public policies. The graph shows the 522 identified papers (125 Pubmed, 397 Scopus) and the 30 selected among them for this review.

## 3. Discussion

The papers selected for this review are summarized in Table 5. The PD cost may be very different from one country to another. Nonetheless, the monetary value of the year in which the study happened should be considered. All values were converted to US dollars ($) and daily, quarterly, or half-yearly results were converted to annual. Only one article had values by period of life [19], and another took into consideration those 40 to 79 years of age [20].

Practically, all articles used the general costs of the disease, without naming the type of economic evaluation. Only one article [21] referred to the burden of disease as DALY (disability-adjusted life in years), suggesting the use of cost-utility.

Because there is no way of implementing measures so as to reduce new cases, the most appropriate model for PD costs may be developed from prevalence studies. They are conducted when diagnosis has already been established, obtaining ample results, and are to conduct than incidence ones, which demand rigorous criteria for diagnosis. In this review, we identified 20 papers that followed this line of research (see Table 4).

The use of questionnaires, suggesting a bottom-up approach, is common practice found in the research reviewed here, although not all of them described the design. The unit value of the inputs used is more easily acquired than full reports obtained from large databases in top-down approaches, although at least six papers suggest the use of this approach for studying large samples [22–27].

Since it is a disease with long survival, retrospective studies are the most common for PD, despite the bias of memory that can be generated depending on the retroactive period. In reviewed articles, 12 authors (see Table 4) opted for a prospective study with patient monitoring. Despite being lengthy and expensive, some studies used Markov models [19] or econometric studies [28, 29] with cohorts, for example.

The aforementioned chosen variables are related to the purpose of the study, but, in general, we observed that 20 (see Table 4) out of 30 papers opted for the total cost of the disease, including direct, indirect, and/or personal costs. In one of the studies [20], however, indirect costs were only considered, from the perspective of insurance companies and human capital, whereas, in another [21], the aim was to evaluate intangible costs alone through lost years of life. Similarly, three studies concentrated only on medication costs [25, 30, 31].

Among direct costs, the most common variables analyzed in most studies were medication, hospitalizations, outpatient visits, auxiliary treatments, home care, transport, and special equipment. Not all studies divided the direct costs into medical and nonmedical (Table 3). In one of the studies, even dental care provided to patients was assessed [29].

Regarding indirect costs, most studies are related to the patient and/or the caregiver in terms of loss of productivity, early retirement, and sick leaves (medical certificates). As for personal costs, they consider informal care, copayment treatments, drugs, and equipment.

The society costs are the most studied, the society being the most affected regarding allocation of resources. Only four studies assessed this prospect from the patient's point of view [27, 32–34].

Clarity is needed in the way data and/or results are expressed, which may generate uncertainty or confusion in the conclusion of a study. For instance, one of the studies [35] does not provide a clear cost of PD, and groups are very stratified and only the differences between them are highlighted. Moreover, albeit the many variables analyzed, important components were not assessed, such as auxiliary treatments. On the other hand, another study [20] does not enlist which direct cost components were used. As such, statistical analyses must be well established, so that other studies can be replicated if necessary. In this review, some studies did not provide that [25, 31, 36].

Many authors have chosen to direct certain types of costs towards one category, which reinforces the uniqueness of each study. One author [37] argued that informal care should be placed with indirect costs, but with direct nonmedical ones, based on the fact that if home care is not provided by the family, professional care would be needed.

The fact that the same cost component is classified in different categories can have a strong influence on the final results, not considering the values set by the inputs in each country. In a study conducted in Russia [37], for example, direct costs accounted for 67% of total costs, while indirect ones accounted for 33%. Besides, in a study in Singapore [38], direct costs were 38.5% and indirect ones 61.5% of the total cost. Another author states [39] that costs were distributed as 35.7% direct, 29.4% as direct nonmedical costs and 34.9% as indirect. In this study, we have verified that the value of consultation with a specialist in the Hungarian public health care system costs around $7, while in the UK [33] this value is approximately $225.

Issues related to health insurance also influence the comparison of studies greatly and must be considered. For example, in India, a study [36] revealed that only 7.4% of patients are covered by health insurance, and, unlike most studies reviewed, the cost of PD treatment is very low, at around $707 per year, since most of the expenses are covered by the patients and their family. Conversely, in the UK [33], maintaining virtually the same research approach, a final value of around $20,000 a year was calculated. In Japan, on the other hand [40], a study found a value of around $6,000 per PD patient, where the health insurance covers 100% of the population. Depending on the patient's income or age, he/she contributes 10% to 30% to medical costs.

There are some other factors that certainly affect the results obtained: samples ranged from small cohorts (*n* = 12) [28] to large populations (*n* = 630,000) [22], some studies [41, 42] have excluded from their samples patients with advanced PD (Hoehn & Yahr 5), and others [19, 43] assessed not only PD, but also its complications and/or comorbidities.

Finally, with regard to the revised articles of this manuscript, we could suggest an instrument as a guideline to determine PD-related costs even though several methodologies and different variables could be taken into account for each particular scenario. Therefore, prospective studies would be the ideal methodology, but cross-sectional, retrospective ones, with a bottom-up approach from the perspective of society, could be more feasible. The questionnaire to obtain data could be divided into the following parts:

1. Clinical, social, demographic, and economic issues of the patient;
2. Medical and nonmedical direct costs;
3. Indirect costs;
4. Personal costs (including caregivers).

The most common variables found in the literature used to determine the costs of PD, depending on the scope of the study, are shown in Table 6.

## 4. Conclusion

The concepts mentioned in this review do not aim to finalize the discussion on health economics tackling the costs of neurodegenerative diseases, such as PD, but only to allow access to introductory concepts of these assessments, so that the reader can contextualize the articles analyzed here.

The very definition of studies on costs of disease suggests limitations, as the articles here reviewed display methodological heterogeneity regarding PD costs, and this variation is an important factor that should receive more attention in literature. Unlike Alzheimer's disease, which has a validated instrument to determine the costs of illness [49], PD presents considerable problems in its analysis, since evaluations and comparisons are made between individual studies. If there were a standardized and validated instrument, the data costs would be more reliable and transparent and there would be rational allocation of resources and better collection of data for cost analysis and efficacy.

We observed that there is no standardization of terminology used for the definition of costs, or even unanimity in the identification of categories because a variable can be found in different classifications, depending on the criterion used by the researcher, which may underestimate the total cost of the disease.

On the other hand, there is no one perfect research methodology covering a single answer for all solutions. Sometimes, certain types of studies are more appropriate than others. There are several limitations that must be discussed and related to, such as the methodological problems, and the validity of their assumptions can differ because they may introduce bias in analysis in favor of a variable, lack of interest in its assessment, or even lack of information. Therefore, researchers must be careful with the source of their data and the method used for performing the calculations, so that their research can be replicated and validated, since there is no specific instrument for the assessment of PD costs. A limitation also deals with the funding sources of studies, which may be from government sources, insurance companies, or pharmaceutical industries, for example, generating important biases that also need to be addressed. It is necessary to define useful metrics for public health and private ones for managers of health, employers, insurance companies, and even patients themselves, because, without this agreement, the work of researchers and the funds invested shall remain uncertain and inconsistent.

Nevertheless, if the evidence obtained is of good quality in terms of transparency, there is quality and credibility in the data completeness of the documentation. Overall, if they are relevant to health care [50], these studies contribute to a better allocation of resources that are not related to savings but evaluate the efficiency, effectiveness, and safety of interventions.

The age group that the PD affects, if well attended to, can experience “healthy aging,” with a good quality of life and preserve its autonomy for longer, thus reducing its cost to the state and society.

## Figures and Tables

**Table 1:** Types of economic evaluation and their main characteristics.

Type of economic analysis	Costs	Advantages	Disadvantages
Cost minimization (CMA)	Monetary	This technique only measures costs	It does not describe results, and it has little applicability to health
Cost-effectiveness (CEA)	Monetary	It allows comparisons between health programs	Difficulty in comparison of results
Cost-benefit (CBA)	Monetary	This analysis allows comparisons between strategies because it works with the same monetary unit	Difficulty of valuing human life
Cost-utility (CUA)	Monetary	This analysis considers the level of well-being and preferences of the individual	The scales of measurement of quality are arbitrary

**Table 2:** Main study designs on costs.

Approaches	Description	Advantages	Disadvantages
Prevalence	Frequency measure It evaluates all existing cases in a given period	Ample results Specific policy planning Fast study and recommended for chronic diseases	Considered weak at estimating the risk of developing disease
Incidence	Frequency measure Assesses the number of new cases in a given period	Implementation of measures to reduce new cases It is used more for acute diseases, since it estimates the risk of developing the disease	Not recommended for chronic diseases
Top-down	It measures the proportion of a disease attributed to several risk factors. It involves a study directed from total to lower levels	When the scope of study is well understood	More comprehensive, it hampers the study on the details of the disease
Bottom-up	Related to the unit costs of inputs used. It involves the study directed from individual levels to the total.	More detailed	Risk of double counting
Prospective	Temporal study, performed during disease. Probes the effect through the cause	Used in chronic diseases	Time-consuming and expensive
Retrospective	Temporal study performed with preexisting data. Probes the cause through the effect	Quick and cheaper	Risk of memory bias
Econometric	Comparison of groups	Minor amount of data required Cost difference between the two populations	Long study, requiring that the control group be paired to the study group
Markov models	Stochastic process Used in prospective studies. Patients stratified in stages of disease	Dynamic model aiming at studying the transition from one stage to another, evaluating the costs of each step	Transition of stages is independent, without considering the previous one

**Table 3:** Classification of costs.

Types of costs	Description
Direct medical	Directly related to the disease. Hospitalization, medication, medical appointments, treatments, laboratory tests, and diagnosis
Direct nonmedical	Directly related to the disease. Transport, domestic modifications, food
Indirect	Loss of productivity: partial, temporary, or permanent They may affect the patient and/or caregiver Early retirement
Intangible	Psychological and psychosocial and costs, difficult measurement
Personal	Costs incurred by the patient and/or their family, when there is no support from private and/or public health care. Private consultations, medication, treatments, and domestic modifications. Linked with direct costs

**Table 4:** Description of the main perspectives used in cost studies.

Perspective	Description
Industry	Related to human capital. Considers the individual as an investment target
Society	More common in the literature. It is comprehensive and based on health-related decisions. It represents the public interest
Patient/family	Less common, only addresses the patient's and their family's costs
Public/private health care	To identify and quantify all inputs used in the production of the service/procedure. Important to form the cost of illness

**Table 5:** Comparison of findings on costs of PD in selected studies.

Author	Country/Region	Year	*n*	Design	Costs studied	Perspective	Value/year US$	Comments
Yoritaka et al. [40]	Japan	2016	715	SPO	D	S	5,828	Direct cost
Martínez-Martin et al., [44]	Spain	2015	174	PO/BU	D/I	S	13,720.24/year 4	Magnitude of disease and quality of life
Tamás et al. [39]	Hungary	2014	110	PE/BU	D/I/OOP	S/CH	6,831	Costs of illness and quality of life
Kowal et al. [22]	USA	2013	630,000	PE	D/I	S	22,800	Economic load current and projected (by 2050) in the USA
Zhao et al. [19]	Singapore	2013	195	PE/MK/BU	D/I	S	68,519 (over the lifetime period)	Cost of illness
Johnson et al. [41]	USA	2013	1,151	RE	D/I	CS	43,506 PDINST (cohort)	Cost of illness x several cohorts
Bhattacharjee and Sambamoorthi [29]	USA	2013	350	RE	D/OOP	S	15,404	Cost of illness/over expenditure associated with PD
Kaltenboeck et al. [23]	USA	2012	25,577	RE	D	G	78,042 (ambulatory pac. PD)	Survival rates and costs of patients of health programs
Bach et al. [43]	Germany	2012	1,449	PE	D/I	G	6.00 (2190) to 12.69 (4631.85)	Cost of illness/drugs/comorbidities
Lökk et al. [25]	Sweden	2012	4,163	PE/RE	D	S	9,333	Cost of illness/drugs
Johnson et al. [20]	USA	2011	278	PO	I	S/CH/CS	569,393 (45 years), 188,590 (55), 35,496 (65), 2,451 (75) (from 40 to 79 years)	Indirect costs
Jennum et al. [26]	Denmark	2011	13,400	RE/PO	D/I	S	7,763	Cost of illness
Zhao et al. [38]	Singapore	2011	195	PE/BU	D/I/OOP	S	10,129	Cost of illness
von Campenhausen et al. [45]	Europe (6 countries)	2011	486	PE/RE/BU	D/I/OOP	S	2,968 to 11,124	Cost of illness
Winter et al. [30]	Italy	2010	70	PO/BU	D/I/OOP	S	19,574	Cost of illness/drugs
Winter et al. [46]	Germany	2010	145	PO/PE/BU	D/I/OOP	G	22,763	Cost of illness
Winter et al. [47]	Germany	2010	145/133	PE/RE	D/I	S	21,138 to 35,864	Cost of illness
Winter et al. [32]	Czech Rep.	2009	100	PE/RE/BU	D/I	S/CH/P	12,483	Cost of illness
Winter et al. [37]	Russia	2009	100	PE/PO/BU	D/I	S/CH	5,935	Cost of illness
Vargas et al. [42]	Brazil	2008	144	PE/PO/BU	IN	NA	NA	Resource use X incapacity
McCrone et al. [33]	UK	2007	175	PE/RE	D/OOP	CS/P	19,861	Cost of illness
Leibson et al. [35]	USA	2006	92	PE/RE	D	NA	Unclear	Cost of illness per groups
Ragothaman et al. [36]	India	2006	175	PE/PO	D	S	707	Cost of illness/direct costs
Wang et al. [48]	China	2006	190	PE/RE/BU	D/I	S	925	Cost of illness
Vossius et al. [31]	Germany/Norway	2006	438	PE/RE/PO	D	S	2,389 (Germany), 1,620 (Norway)	Cost of PD drugs
Noyes et al. [27]	USA	2006	717	PE/RE	D/OOP	S/P	18,528	Cost of illness/drugs/medicare
Cordato et al. [28]	Australia	2006	12	PE/PO	D/I	S	5,380	Cost of illness
Huse et al. [24]	USA	2005	20,016	PE/RE	D	CS	10,037	Cost of illness
Spottke et al. [34]	Germany	2005	145	PE/PO	D/I/OOP	S/G/P	22,723 ± 28,297	Cost of illness
Cubo et al. [21]	Spain	2005	23,417	RE	Int.	G	NA	Years of life lost

Notes: SPO = semiprospective; PO = prospective; BU = bottom-up; PE = prevalent, MK = Markov; RE = retrospective; D = direct cost; I = indirect cost; OOP = out-of-pocket; Int. = intangible; S = society; CH = human capital; CS = insurance companies; G = government; NA = not applicable; P = patient; PDINST = patients with PD institutionalized; Medicare = USA health care.

**Table 6:** Most common variables found in the cost studies of Parkinson's disease.

Patient/disease	Direct medical cost	Direct nonmedical cost	Indirect cost	Out-of-pockets
Age	Hospitalization	Ancillary therapy/rehabilitation	Retirement	Transportation^*∗*^
Gender	Pharmacotherapy (PD and comorbidities)	Home Care^*∗*^	Retirement premature	Special food
Marital status	Outpatient visit	Transportation^*∗*^	Sick leave	Laundry
Instruction	Diagnostics	Special equipment^*∗*^	Working days loss of the patient	Home Care^*∗*^
Working status	Nursing home	Home modification^*∗*^	Working days loss of the caregivers	Caregivers
Duration of PD		Copayments^*∗*^	Productivity loss	Special equipment^*∗*^
Comorbidities			Loss of leisure time	Home modification^*∗*^
H & Y stage^1^				Private health plans
UPDRS^2^				Copayments^*∗*^
PQD-39^3^				
MMSE^4^				

^1^Hoehn & Yahr scale of disability/^2^Unified Parkinson's Disease Rating Scale/^3^Parkinson's Disease Questionnaire–39 (quality of life)/^4^Mini-Mental State Examination.

^*∗*^Variables that may be in more than one cost type.
